# Single-Atom Catalysts for Electrochemical Hydrogen Evolution Reaction: Recent Advances and Future Perspectives

**DOI:** 10.1007/s40820-019-0349-y

**Published:** 2020-01-13

**Authors:** Zonghua Pu, Ibrahim Saana Amiinu, Ruilin Cheng, Pengyan Wang, Chengtian Zhang, Shichun Mu, Weiyue Zhao, Fengmei Su, Gaixia Zhang, Shijun Liao, Shuhui Sun

**Affiliations:** 1 Institut National de la Recherche Scientifique-Énergie Matériaux et Télécommunications, Varennes, QC J3X 1S2 Canada; 2 State Key Laboratory of Advanced Technology for Materials Synthesis and Processing, Wuhan University of Technology, Wuhan, 430070 People’s Republic of China; 3 The Key Laboratory of Fuel Cell Technology of Guangdong Province, The Key Laboratory of New Energy Technology of Guangdong Universities, School of Chemistry and Chemical Engineering, South China University of Technology, Guangzhou, 510641 People’s Republic of China; 4 Key Laboratory of Materials Processing and Mold, Ministry of Education, Zhengzhou University, Zhengzhou, 450002 People’s Republic of China

**Keywords:** Single-atom catalysts, Nanomaterials, Electrocatalyst, Hydrogen evolution reaction, Electrochemical energy conversion

## Abstract

Hydrogen, a renewable and outstanding energy carrier with zero carbon dioxide emission, is regarded as the best alternative to fossil fuels. The most preferred route to large-scale production of hydrogen is by water electrolysis from the intermittent sources (e.g., wind, solar, hydro, and tidal energy). However, the efficiency of water electrolysis is very much dependent on the activity of electrocatalysts. Thus, designing high-effective, stable, and cheap materials for hydrogen evolution reaction (HER) could have a substantial impact on renewable energy technologies. Recently, single-atom catalysts (SACs) have emerged as a new frontier in catalysis science, because SACs have maximum atom-utilization efficiency and excellent catalytic reaction activity. Various synthesis methods and analytical techniques have been adopted to prepare and characterize these SACs. In this review, we discuss recent progress on SACs synthesis, characterization methods, and their catalytic applications. Particularly, we highlight their unique electrochemical characteristics toward HER. Finally, the current key challenges in SACs for HER are pointed out and some potential directions are proposed as well.

## Highlights

- All the important single-atom catalysts (SACs) synthetic strategies, such as wet-chemistry method, atomic layer deposition, metal–organic framework-derived method, electrodeposition, high-temperature atom trapping from bulk particles, and vacancies/defects immobilized strategy, have been summarized and discussed in detail.
- Various metal-based (especially Pt, Pd, Ru, Fe, Co, Ni, Mo, W, V) SACs in electrocatalytic hydrogen evolution reaction (HER) have been systematically reviewed.
- The current key challenges in SACs for electrochemical HER are pointed out, and some potential strategies/perspectives are proposed.

## Graphical abstract

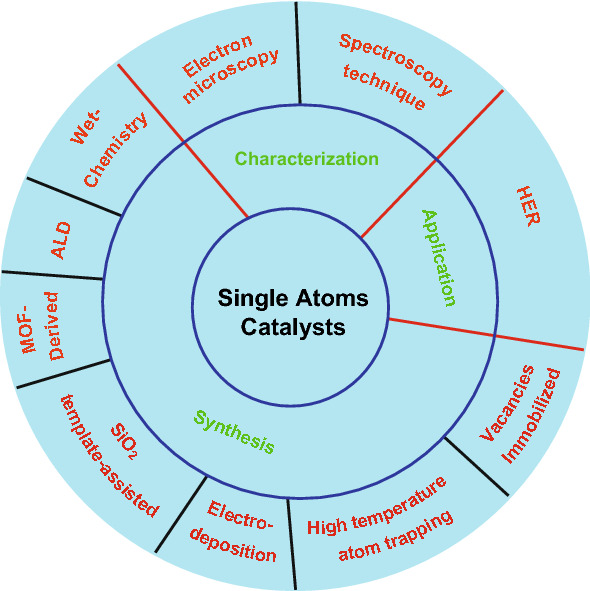

## Introduction

With the depletion of fossil fuels (coal, oil, and natural gas), heavily environmental pollution, and climate change, the exploitation of safe, clean, efficient, sustainable, and environmental-friendly energy sources has become a major societal and technological pursuit in the twenty-first century [1–7]. Hydrogen is considered as a viable substitute for fossil fuels in the future because of its renewability, zero carbon dioxide emission, and high mass-specific energy density [8–12]. Among the hydrogen production method, water electrolysis is an attractive way due to its device simplicity, high product purity, and renewability [13–17]. The process of water electrolysis is based upon two half-reactions: one reaction is oxygen evolution reaction (OER), and the other reaction is hydrogen evolution reaction (HER) [18–28]. Although water electrolysis has received much attention, the implementation of efficient water splitting technology is still a huge challenge due to the kinetic barrier of both reactions. In fact, both HER and OER suffer from high overpotentials or low Faradic yields for electrodes which lead to low energy utilization [29–40]. In order to overcome these problems, appropriate catalysts are needed to improve electrode efficiency by decreasing the activation energy and increasing the conversion efficiency. In other words, catalysts materials play an important role in improving the performance of both HER and OER. Currently, Pt group electrocatalysts are regarded as the benchmark for HER because they exhibit excellent activity over the pH range from 0 to 14 [41–53]. Nevertheless, the high cost, poor stability, and the low availability of noble metal limit their wide applications. Therefore, it is highly urgent to develop low-cost, highly active, and sustainable electrocatalytic materials for HER. For this purpose, extensive efforts have been devoted to preparing low-Pt even non-Pt-based electrocatalytic materials [54–72].

Single-atom catalysts (SACs), with only isolated single-atom dispersion on the support surface, have attracted extensive attention in many kinds of catalysis community recently due to their maximum atom-utilization efficiency, high selectivity, and unique properties [73–80]. The first reported SACs by Zhang’s groups occurred in 2011 where a coprecipitation method was employed to synthesize Pt SACs on iron oxide substrate (Pt_1_/FeO_*x*_), showing high performance and durability toward the CO oxidation [74]. Compared with other heterogeneous materials, SACs not only possess homogenized active species for catalytic reactions which are similar to homogeneous materials, but also have extra advantages of high reusability and durability that originate from heterogeneous materials. Therefore, SACs have great advantages [73, 81–86]. Given this unique characteristic, SACs have attracted extensive attention in various kinds of catalytic applications, including HER, OER, organic catalytic reaction, oxygen reduction reaction (ORR), N_2_ reduction reaction (NRR), CO_2_ reduction reaction (CO_2_RR), and other important reactions [74, 87–123]. In addition, advanced characterization techniques including scanning tunneling microscope (STM), aberration-corrected high-angle annular dark-field scanning transmission electron microscopy (AC-HAADF-STEM), synchrotron-radiated X-ray absorption fine structure (XAFS) spectroscopy, etc., are widely adopted for the characterization of SACs, which can directly measure the single atom to confirm the structure and electronic properties of SACs including a confirmation of the single metal atom, the chemical state of the metal center, and the coordination environment [121, 124]. Furthermore, density functional theory (DFT) simulation has brought unprecedented to discovering catalytic reaction mechanisms, enabling the rational design of materials with tailored activity.

In this review, we focus on SACs toward HER, with a much more comprehensive and detailed introduction and discussions. We first highlight several novel synthetic methods, especially the atomic layer deposition (ALD), the metal–organic framework (MOF)-derived strategy, and vacancies/defects immobilized methodology, for SACs synthesis. Next, to reveal the structures and compositions of SACs, different characterization techniques, such as XAFS spectroscopy, AC-HAADF-STEM techniques, and DFT simulation, have been summarized and discussed. In addition, Pt, Pd, Ru, Fe, Co, Ni, Mo, W, V, etc., metal-based SACs in electrocatalytic HER have been systematically reviewed. Finally, the current key challenges in SACs for electrochemical HER are pointed out and some potential strategies/perspectives are proposed as well. Thus, this review covers the key aspects of SACs for hydrogen production via HER, including key synthesis strategies, evidence-based characterizations, and impact in the field of hydrogen production. We believe it is of great importance and interest to the development of SACs toward the application of HER, and it can be extended to other fields as well.

## Synthetic Strategies

In general, it is still a huge challenge to prepare SACs with robust structures and high performance due to its aggregation tendency. Fortunately, technological advances have evolved with effective methods to overcome such difficulties. In this rapidly developing area, some methods have been exploited for constructing SACs, including the wet-chemistry method, ALD, MOF-derived method, SiO_2_ template-assisted pyrolysis method, electrodeposition approach, high-temperature atom trapping method, vacancies/defects immobilized strategy, photochemical reduction, iced photochemical reduction, chemical reduction, hydrothermal reduction methods, and ambient synthetic strategy [52, 53, 74, 93, 125–127]. In addition, some advantages and disadvantages of these synthesis strategies for preparing SACs are listed in Table 1.

**Table 1:** Partial list of synthesis strategies for producing SACs

Synthetic method	Advantage	Disadvantage	References
Wet-chemistry method	Simply equipment; easily large-scale production	Low metal loading	[74, 123, 129]
ALD	Precise control SACs on different substrates	Low yields; high equipment cost	[52, 137, 139]
MOF-derived method	Easily introduce heteroatoms to anchoring metal atoms	High synthesis temperature	[39, 103, 150–152]
SiO_2_ template-assisted pyrolysis	Potential large-scale production	Dangerous etching reagent (HF)	[107, 148, 149]
Electrodeposition	Controllable amount and size; generally deposited on the outermost surface; facile	Non-uniform plating	[53, 93]
High-temperature atom trapping from bulk particles	Potential large-scale production	High synthesis temperature	[156–159]
Vacancies/defects immobilized	Easily capture or anchor metal species	Non-stability of the defects	[161–163]
Iced photochemical reduction	Tuning the solid nucleation	A sluggish nucleation rate	[125]
Ambient synthetic strategy	Eco-friendly; low cost; mass production of SACs	Non-general method	[127]

### Wet-Chemistry Strategies

Generally, the wet-chemistry routes to prepare SACs involve three steps. The first involves the introduction of the metal species on various supports via impregnation and coprecipitation. The second concerns drying and annealing, and the third is by reduction or activation [73]. In theory, SACs can be obtained by reducing the metal species loading to a fairly low level. However, on the one hand, in most catalytic reactions, more accessible catalytic active species are required. On the other hand, along with the reduced metal, the smaller particle size can also result in increased surface energy, thereby making the metal atoms easily aggregate into larger particles. Therefore, for the wet-chemistry approach, SACs require to be effectively embedded in the support in order to avoid aggregation of the metal atoms into metal nanoclusters or even large nanoparticles [121].

**Fig. 1:**
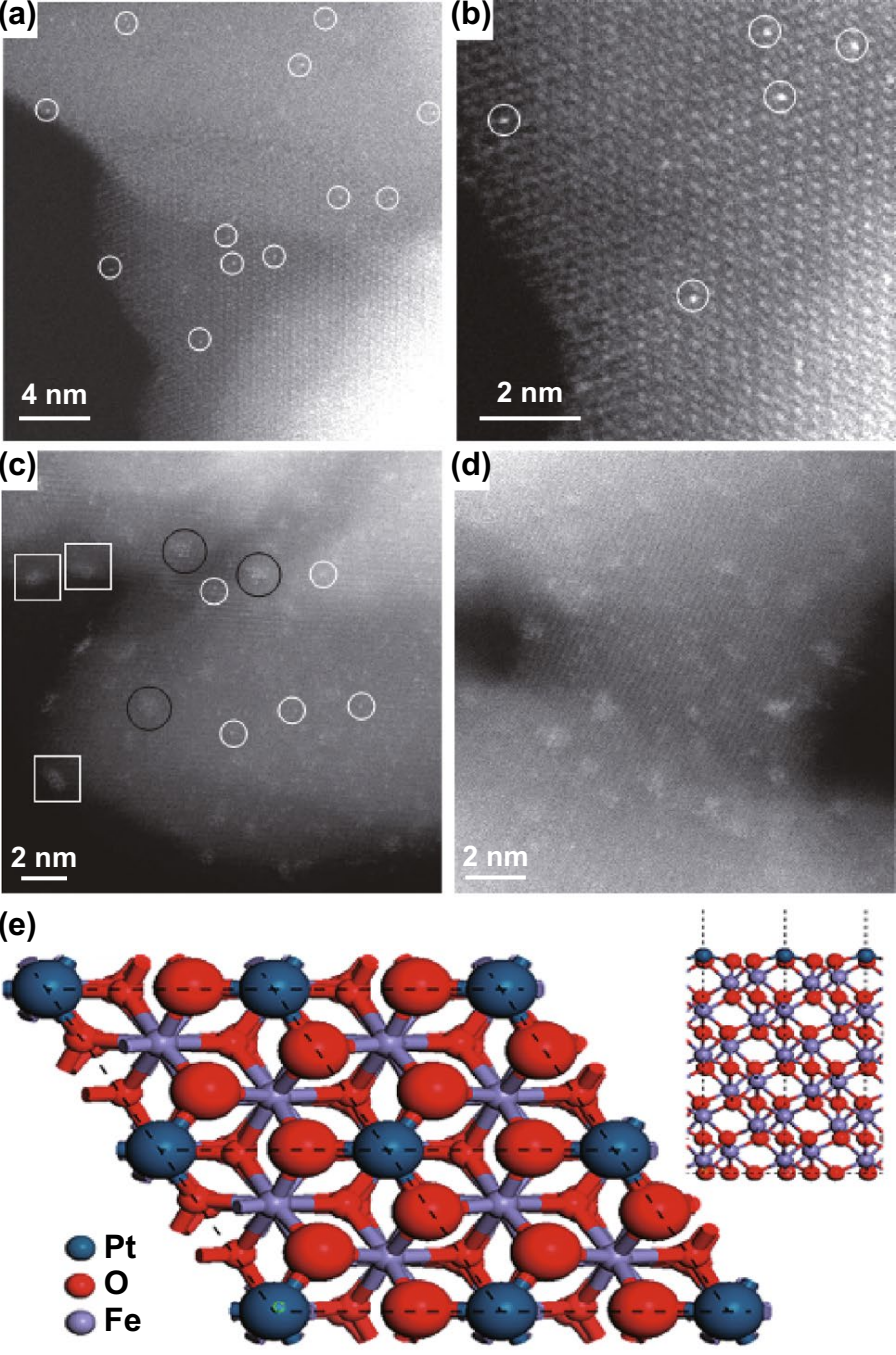
**a** Pt SACs are uniformly distributed on the FeO_*x*_ and **b** directly occupy the positions of the Fe atoms. **c**, **d** A mixture of SACs (in white circles), 2D Pt rafts consisting of less than ten Pt atoms (in black circles) and the size of 3D Pt clusters about 1 nm or less (white squares) are seen clearly. **e** DFT model of Pt_1_/FeO_*x*_. **a**–**e** Reproduced from Ref. [77] with permission. Copyright 2011 Nature Publishing Group

The wet-chemistry strategy has been recognized as an effective method for the synthesis of metal single atoms supported on various oxide substrates. An added advantage of this method is that no specialized equipment is needed. Additionally, it is also the preferred method for potential large-scale production of supported metal catalysts. However, the wet-chemistry strategy has obvious disadvantages of preparing high metal loading materials [128]. For example, Zhang’s group successfully developed Pt SACs supported on FeO_*x*_ (Pt_1_/FeO_*x*_) by the coprecipitation method with a Pt loading only ~ 0.18 wt%. The HAADF-STEM images of Pt_1_/FeO_*x*_ further confirm the presence of Pt single atoms (Fig. 1a–d). In addition, DFT simulation (Fig. 1e) indicates that the most probable sites for Pt SACs are located on the O_3_-terminated surface. In other words, each Pt single atom is coordinated with three surface O atoms [77]. To date, all kinds of oxides substrate such as FeO_*x*_, TiO_2_, CeO_2_, Al_2_O_3_, and ZnO have been investigated as support materials for SACs of Pt, Ir, Rh, Au, and Pd [74, 129–134]. Such catalysts have displayed high catalytic performance toward the water–gas shift reaction, CO oxidation, and selective organic conversion reactions.

### Atomic Layer Deposition Method

**Fig. 2:**
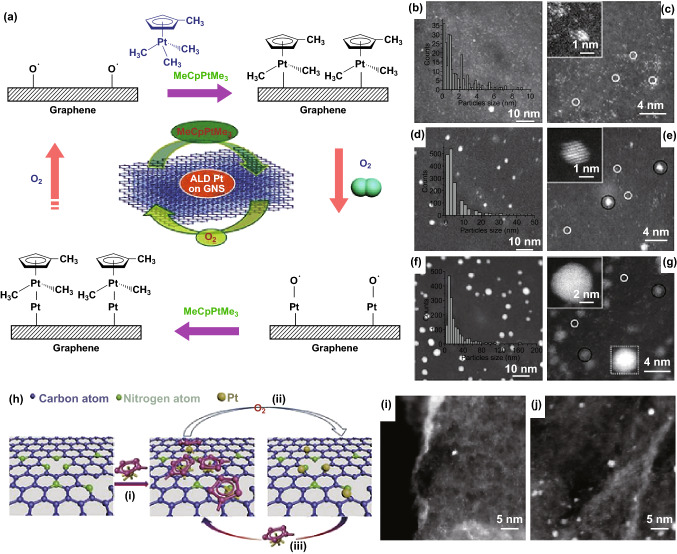
**a** Schematic illustrations of Pt ALD mechanism on graphene nanosheets. **b**–**g** Present the results with (**b, c**) 50, (**d, e**) 100, and (**f, g**) 150 ALD cycles. **h** Schematic illustration of the Pt ALD mechanism on NGNs. HAADF-STEM images of ALDPt/NGNs samples with **i** 50 and **j** 100 ALD cycles. **a**–**g** Reproduced from Refs. [137, 52] with permission. Copyright 2013 and 2016 Nature Publishing Group

In general, the ALD technique has been used to prepare metal oxide thin films with atomically precise control [135, 136]. In 2013, the ALD technique was adopted to fabricate Pt SACs for the first time by Sun’s group [137]. As illustrated in Fig. 2a, the implanted oxygen atoms on the surface of graphene nanosheets can react with (methylcyclopentadienyl) trimethylplatinum (MeCpPtMe_3_), in which some of the organic linkers are converted into H_2_O, CO_2_, and hydrocarbon fragments, leading to the formation of Pt-containing monolayers. Next, the Pt-containing monolayer reacts with subsequent O_2_ to form a new absorbed oxygen layer on the Pt surface. This two-step process forms a whole ALD cycle. The morphology, size, and loading weight of Pt SACs can be adjusted by changing the number of ALD cycles (Fig. 2b–g). Using a similar method, Cheng and coworkers also prepared Pt SACs on the nitrogen-doped graphene (Fig. 2h). As shown in Fig. 2i, j, the size and loading density of the Pt catalyst can be well adjusted through changing the ALD cycles. More importantly, the obtained N-doped graphene nanosheets-supported Pt SACs exhibit excellent HER catalytic activity [52]. Although using the ALD method, some noble metal Ru, Pt, and Pd SACs can be successfully grown on several types of substrates, such as SiO_2_, Al_2_O_3_, and TiO_2_ [52, 137–140]. It still suffers from low yields, high cost of equipment, and precursors, which is not favorable for widespread production [141–143].

### MOF-Derived Method

**Fig. 3:**
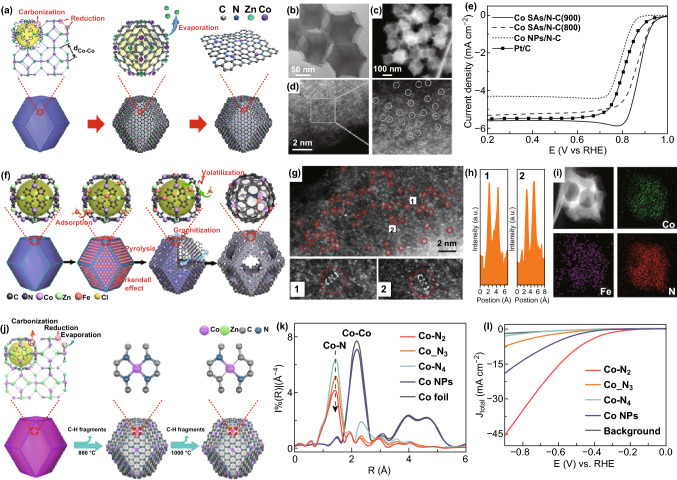
**a** The schematic illustrations of Co SAs/N–C. **b** TEM, **c, d** HAADF-STEM images of Co SAs/N–C(800). **e** LSV of Co SAs/N–C, Co NPs-N/C, and Pt/C in O_2_-saturated 0.1 M KOH solutions. **f** Preparation of (Fe, Co)/N–C. **g** HAADF-STEM of (Fe, Co)/N–C. **h** Corresponding intensity profiles obtained on the zoomed areas in **g**. **i** Corresponding EELS mapping of Co, Fe, and N. **j** Schematic formation process of Co–N_4_ and Co–N_2_. **k** EXAFS spectra confirm the atomic dispersion of Co atoms in Co–N_2_, Co–N_3_ and Co–N_4_. **l** LSVs of Co–N_2_, Co–N_3_, Co–N_4_ and Co NPs. **a**–**e** and **j**–**l** Reproduced from Refs. [102, 147] with permission. Copyright 2016 and 2017 Wiley-VCH. **f**–**i** Reproduced from Ref. [150] with permission. Copyright 2017 American Chemical Society

Metal–organic frameworks (MOFs), due to their high specific surface area, tunable porosity, and unique structures, have attracted enormous attention for many applications including catalysis, sensing, separation, and gas adsorption [144–147]. Researchers have obtained different functional MOFs by changing the metal ions and organic precursors. Very recently, a series of single atoms of Co, Fe, Ni, and W anchored on nitrogen-doped carbon frameworks have been fabricated by pyrolysis MOFs [39, 89, 148, 149]. Li and coworkers first reported that single Co atoms can be obtained by pyrolysis Zn/Co bimetallic zeolitic imidazolate framework (ZIF). Figure 3a illustrates the formation mechanism of Co SAs/N–C. During annealing, the Zn/Co bimetallic ZIF and the organic precursors of ZIFs are transformed into nitrogen-doped porous carbon (N–C). Subsequently, the Zn and Co ions are reduced by the N–C. Pre-meditated mixing of Zn can not only change the distance of adjacent Co atoms and provide N-rich centers, but also avoid the formation of Co–Co bonds under the high temperatures (~ 900 °C). Finally, the Co single atoms (Co SAs) anchored on the N–C can be obtained after evaporation of low boiling point Zn atoms [39]. The HAADF-STEM images of the obtained Co SAs/N–C are illustrated in Fig. 3b–d. Electron energy loss spectrum (EELS) and X-ray absorption spectroscopy (XAS) characterization further confirm the formation of Co–N_4_ structure. Interestingly, such Co–N_4_ structure shows outstanding ORR catalytic activity with a 0.88 V half-wave potential (Fig. 3e). Similarly, Fe–Co dual SACs anchored on N–C have also been prepared by pyrolysis Fe/Co bimetallic ZIF (Fig. 3f–i) [150]. Additionally, the coordination number of Co SAs can be well adjusted by varying the pyrolysis temperature of Zn/Co bimetallic ZIF. For example, different Co–N coordination numbers of Co–N_2_, Co–N_3_, and Co–N_4_ can be selectively prepared at different pyrolysis temperatures (1000, 900, and 800 °C) (Fig. 3j, k). These Co–N_2_, Co–N_3_, and Co–N_4_ structures have been also applied as effective CO_2_RR catalysts. As exhibited by the polarization curves in Fig. 3l, Co–N_2_ achieves the excellent catalytic activity toward CO_2_RR [147].

The molecular-scale cavities within MOFs are usually interconnected by pores. Thus, metal species with an appropriate size can be encapsulated and separated in the cages. For example, Fe(acac)_3_ has a molecular diameter of ca. 9.7 Å, larger than the pore size (3.4 Å) but lower than the cavity diameter (11.6 Å) of ZIF-8. When ZIF-8 is mixed with the Fe(acac)_3_ molecules, the Fe(acac)_3_ molecules can be encapsulated by the ZIF-8 cages. After the pyrolysis of the confined Fe(acac)_3_, ZIF-8 is converted to N–C. At the same time, Fe(acac)_3_ is reduced by the generated N–C, leading to the formation of Fe SACs on the N–C [103]. Replacement of Fe(acac)_3_ with Ru_3_(CO)_12_ produces isolated Ru_3_ clusters embedded on N–C by the same synthesis strategy [151]. In 2018, Cheng et al. [148] reported a novel strategy to construct W SACs on N–C via using MOFs as the precursor. It can be noticed that the uncoordinated amine groups in the UiO-66-NH_2_ are crucial for avoiding the aggregation of W species. Oppositely, the W atoms tend to aggregate into nanoclusters or even large nanoparticles without the dangling ‒NH_2_ groups. The obtained W SACs exhibited good HER activity under alkaline conditions.

This synthesis approach has been extended for the preparation of other SACs (e.g., Ru SACs) with tailored catalytic reaction properties [152]. Therefore, the recent progress indicates that MOFs have several advantages as templates of SACs. First, different kinds of metal ions are bridged via different organic precursors, ensuring the formation of various functional SACs by pyrolysis MOFs. Second, organic linkers derived heteroatom-doped carbon by pyrolysis can be anchored on SACs [82].

### SiO_2_ Template-Assisted Pyrolysis Method

**Fig. 4:**
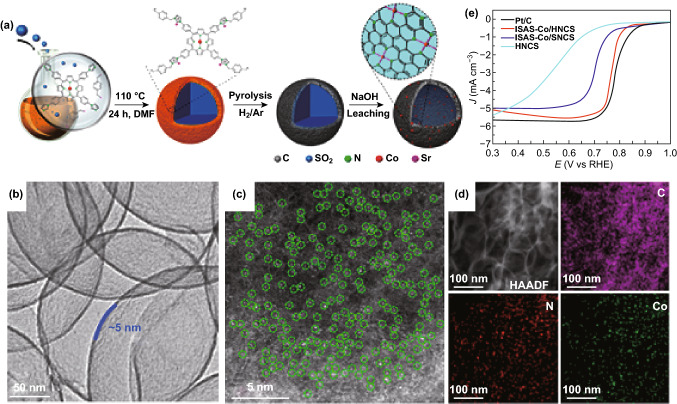
**a** The synthetic process of ISAS-Co/HNCS. **b** TEM and **c** AC-HAADF-STEM images of ISAS-Co/HNCS. **d** HAADF-STEM image and corresponding EDX element mapping of ISAS-Co/HNCS. **e** ORR polarization curves. **a**–**e** Reproduced from Ref. [107] with permission. Copyright 2017 American Chemical Society

**Fig. 5:**
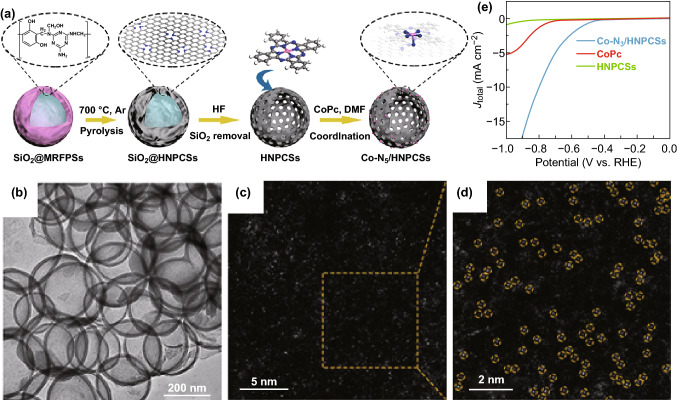
**a** Schematic illustration, **b** TEM, **c** HAADF-STEM image of Co-N_5_/HNPCSs. **d** AC-HAADF-STEM and magnified images of Co-N_5_/HNPCSs. **e** LSV curves. **a**–**e** Reproduced from Ref. [149] with permission. Copyright 2018 American Chemical Society

The use of SiO_2_ as a template is another method for the synthesis of SACs and was recently reported by Li’s group. Briefly, as schematically illustrated in Fig. 4a, a SiO_2_ template is first fabricated and dissolved in a Co-TIPP/TIPP solution before introducing another precursor. Next, the obtained powder is thermally treated under the H_2_/Ar. Finally, the Co SACs can be collected after removing the SiO_2_ template by HF (or NaOH) solutions. AC-HAADF-STEM along with EELS further demonstrates that the formation of Co SACs and the C, N, Co atoms are uniformly distributed (Fig. 4b–d) [107]. Using a similar approach, Mo, Cu, Pt, and Pd SACs have been also fabricated by the same group with SiO_2_, chitosan, and metal salts as precursors. Particularly, the structure of the Mo SACs catalyst was probed by AC-HAADF-STEM and XAFS which indicated the formation of Mo SACs anchored to two carbon atoms and one nitrogen atom (Mo_1_N_1_C_2_) [153]. As shown in Fig. 5a–e, the Co SACs with five Co–N coordination number embedded in polymer-derived hollow N–C spheres (Co-N_5_/HNPCSs) have been also prepared by this method [148]. However, the SiO_2_ template-assisted pyrolysis method would not be a preferred technique in the industry due to the dangerous nature of HF as a dissolution reagent.

### Electrodeposition Approach

Various metal ions can be reduced through the electrochemical cathodic reduction method. The deposition rate of metal ions on the substrate can be adjusted via a change in ion concentration, and then a slow metal ion diffusion rate enables the deposition of SACs on support materials. It is confirmed that Pt atoms can be dissolved from Pt anodes under both alkaline and acid solutions at high voltages and then redeposited onto cathodes [154]. Based on this phenomenon, Pt foil was utilized as a metal source for depositing Pt SACs supported on CoP nanotube arrays by Luo’s group. The obtained Pt SACs supported on CoP nanotube arrays can be directly served as HER electrocatalyst, which exhibited activities comparable to commercial Pt/C in neutral solutions (pH = 7.2) [53]. Similarly, Pt SACs on the single-wall CNTs can be also obtained by a simple electroplating deposition method [93]. In addition, Xue et al. [155] found that Ni and Fe SACs can be obtained by an electrodeposition method. In their work, they declared that Ni SACs (1.23 Å) and Fe SACs (1.02 Å) were obtained and these SACs exhibited high HER performance with close to 0 mV onset overpotential, low Tafel slopes, and large turnover frequencies. As illustrated in the experiments, the electrodeposition method for SACs has several advantages: First, the particle size can be well adjusted by changing the deposition parameters such as plating time. Second, the SACs are usually deposited onto the outside surface of the substrate, which is beneficial for the exposure of the active sites. Third, the electrochemical deposition method is fast, scalable, controllable, and efficient [34, 82]. However, there are also disadvantages for this electrodeposition process, such as non-uniform plating (electroplating that results in a substandard appearance of the plated material).

### High-Temperature Atom Trapping from Bulk Particles

**Fig. 6:**
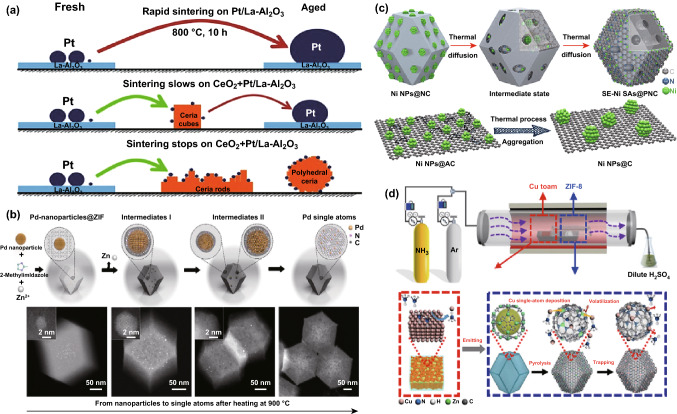
**a** Schematic illustration of Pt nanoparticle to Pt SACs. **b** Schematic illustration of Pd nanoparticle to Pd SACs and structural characterizations of Pd SACs. **c** Scheme of Ni NPs to Ni SACs transformation and structural characterizations. **d** Schematic of the synthesis of Cu SACs/N–C. **a** Reproduced from Ref. [156] with permission. Copyright 2016 American Association for the Advancement of Science. **c** Reproduced from Ref. [158] with permission. Copyright 2018 Wiley-VCH. **b**, **d** Reproduced from Refs. [157, 159] with permission. Copyright 2018 Nature Publishing Group

In the high-temperature atom trapping process, a thermal transport from bulk nanoparticles to the SACs is realized by a simple model system. This approach not only requires the mobile metal species, but also demands a support material that can capture the mobile metal species. As illustrated in Fig. 6a, under high temperatures and oxidizing conditions, Pt atoms can be emitted to PtO_2_ molecules [156]. When PtO_2_ molecules bind to the surfaces of another material that stabilizes the metal-containing precursors, then uniformly dispersed metal SACs can be obtained. Indeed, Datye’s group described the preparation of Pt SACs supported on CeO_2_ by thermal diffusion of Pt nanoparticles [156]. Recently, Li’s group reported that precious metal (Au, Pd, and Pt) nanoparticles can be converted to thermally stable noble metal single atoms (Au, Pd, and Pt SACs) by utilizing ZIF-8 derived N–C as the anchoring substrate to capture the migrating noble metal atoms at 900 °C (Fig. 6b) [157]. Interestingly, this phenomenon is existed in non-precious metal system as well. For example, Ni nanoparticles supported on the defective N–C can be converted to surface enriched Ni SACs. The authors declared that the Ni nanoparticles play a part of ‘‘Pac-Man’’ to bite off surface C–C bonds. Meanwhile, when Ni nanoparticles diffuse on the N–C matrix, the metal Ni atoms bound to the N-rich defect sites (Fig. 6c). Therefore, Ni nanoparticles are slowly worn and finally converted to Ni SACs [158].

Furthermore, in order to find a feasible strategy for producing functional SACs at industrial levels, Qu et al. described a facile gas-migration method to directly convert bulk metal materials to SACs. The schematic is shown in Fig. 6d. First, ZIF-8 and commercial Cu foam are placed separately in a porcelain boat. Second, ZIF-8 is subjected to a pyrolysis process at 900 °C under Ar atmosphere, forming pyrolyzed ZIF-8 with empty Zn nodes and a large number of defect sites. Subsequently, under NH_3_ atmosphere, NH_3_ molecules haul the metal Cu atoms from the surface of Cu foam to form volatile Cu(NH_3_)_*x*_ species. If such Cu(NH_3_)_*x*_ species bind to the defects-rich N–C support, then Cu SACs can be uniformly dispersed on the surface of the N–C support [159]. These high-temperature atom trapping methods provide valuable guidance for the direct preparation of SACs from non-precious bulk metals (Cu, Ni, and Co) and show great potential for scaling up SACs toward industrial applications. Additionally, using bulk noble metal (Pt net, Au plate, and Pd plate) as a precursor, Pt SACs, Au SACs, and Pd SACs can be also trapped by the defective graphene (DG). Along with them, the as-obtained Pt SACs/DG shows high activity for the HER [160].

### Vacancies/Defects Immobilized Strategy

SACs trapped by defect sites in various substrates (2D materials and transition metal compounds) form a unique class of single-atom catalysts. For example, Wang’s group reported the electrochemical exfoliation Mo_2_TiAlC_2_ MXene with Pt plate as the counter electrode, in which the Mo vacancies can use as the anchoring sites for Pt SACs (Mo_2_TiC_2_T_*x*_–PtSA). During the electrochemical exfoliation process, single Pt atoms are simultaneously immobilized on the Mo vacancies and stabilized via the formation of covalent Pt–C bonds with the surrounding C atoms on the MXene. The resultant Mo_2_TiC_2_T_*x*_–PtSA materials show Pt-like activity with only 30 mV overpotential at 10 mA cm^−2^ toward HER [161]. Similarly, Chen’s group reported a general and facile synthesis approach to fabricate a series of SACs by a simultaneous self-reduction-stabilization process under ambient conditions using 2D Ti-vacancy-rich Ti_3−*x*_C_2_T_y_ MXene nanosheets as support. The series of precious and non-precious metal (M) single atoms (M = Pt, Ru, Rh, Ir, Pd, Fe, Co, and Ni) can be fabricated through the formation of M–C bonds [162]. Besides the defect-rich 2D MXene used as substrates to immobilize the SACs, other vacancies/defects-rich materials also have been used to stabilize SACs, such as vacancy-rich nickel hydroxide [163], oxygen vacancies-rich MoO_2_ [164], oxygen vacancies on ceria [165].

### Others Synthetic Approach

**Fig. 7:**
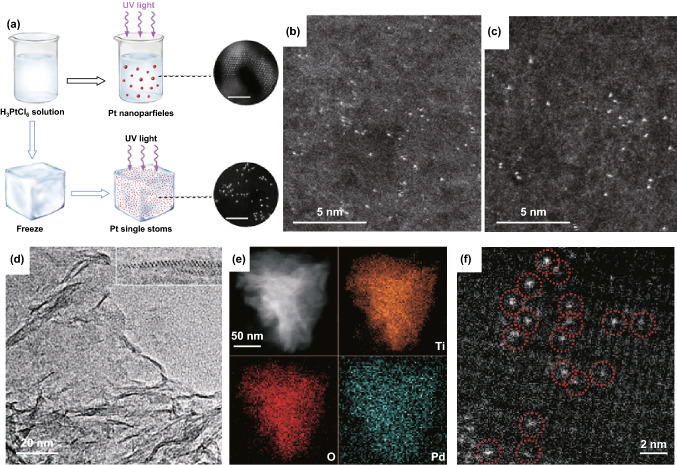
**a** Schematic illustration of the iced photochemical process. AC-HAADF-STEM images of **b** Ag SACs and **c** Au SACs. **d–f** Structural characterizations of Pd_1_/TiO_2_. **a–c** Reproduced from Ref. [125] with permission. Copyright 2015, Royal Society of Chemistry. **d**–**f** Reproduced from Ref. [84] with permission. Copyright 2016 Science

In addition to the above-mentioned synthetic strategy for SACs, many other methods have also been reported by different research groups, including hydrothermal method, iced photochemical reduction, photochemical reduction, and chemical reduction. In particular, Bao’s group demonstrated that Pt SACs supported on MoS_2_ can be obtained by a hydrothermal reduction method. Such materials exhibit an improved HER performance compared to the original MoS_2_. By combining DFT calculations, they declared that the improved HER performance comes from the tuned hydrogen adsorption free energy [92]. Wei et al. also reported a facile method to fabricate Pt SACs by photochemical reduction of frozen H_2_PtCl_6_ solution under ultraviolet light irradiation (Fig. 7a). The aggregation of Pt atoms can be avoided through iced photochemical reduction, and therefore, Pt SACs are obtained successfully. Furthermore, the Pt SACs can be deposited on various kinds of substrates (such as ZnO nanowires, TiO_2_ nanoparticles, and carbon materials) [125]. Particularly, the Pt SACs on mesoporous carbon showed excellent HER performance with an overpotential of 65 mV at 100 mA cm^−2^ and long-time durability against commercial Pt/C. More importantly, the iced photochemical reduction method can be applied to synthesis Au (Fig. 7b) and Ag SACs (Fig. 7c). In addition, Zheng’s group prepared Pd SACs on ultrathin titanium oxide nanosheets with a high Pd loading (1.5 wt%) by a room-temperature photochemical reduction method (Fig. 7d–f) [84].

## Characterization Technology

In order to confirm the structures and compositions of SACs, a variety of atomic resolution characterization and analytical techniques have been employed, including AC-HAADF-STEM, XAFS spectroscopy [X-ray absorption near-edge structure (XANES) and extended X-ray absorption fine structure (EXAFS)], infrared (IR) spectroscopy, and nuclear magnetic resonance (NMR). In addition, DFT computations have brought unprecedented to discovering catalytic reaction mechanism and predicting the catalytically active species.

### Electron Microscopy Techniques

**Fig. 8:**
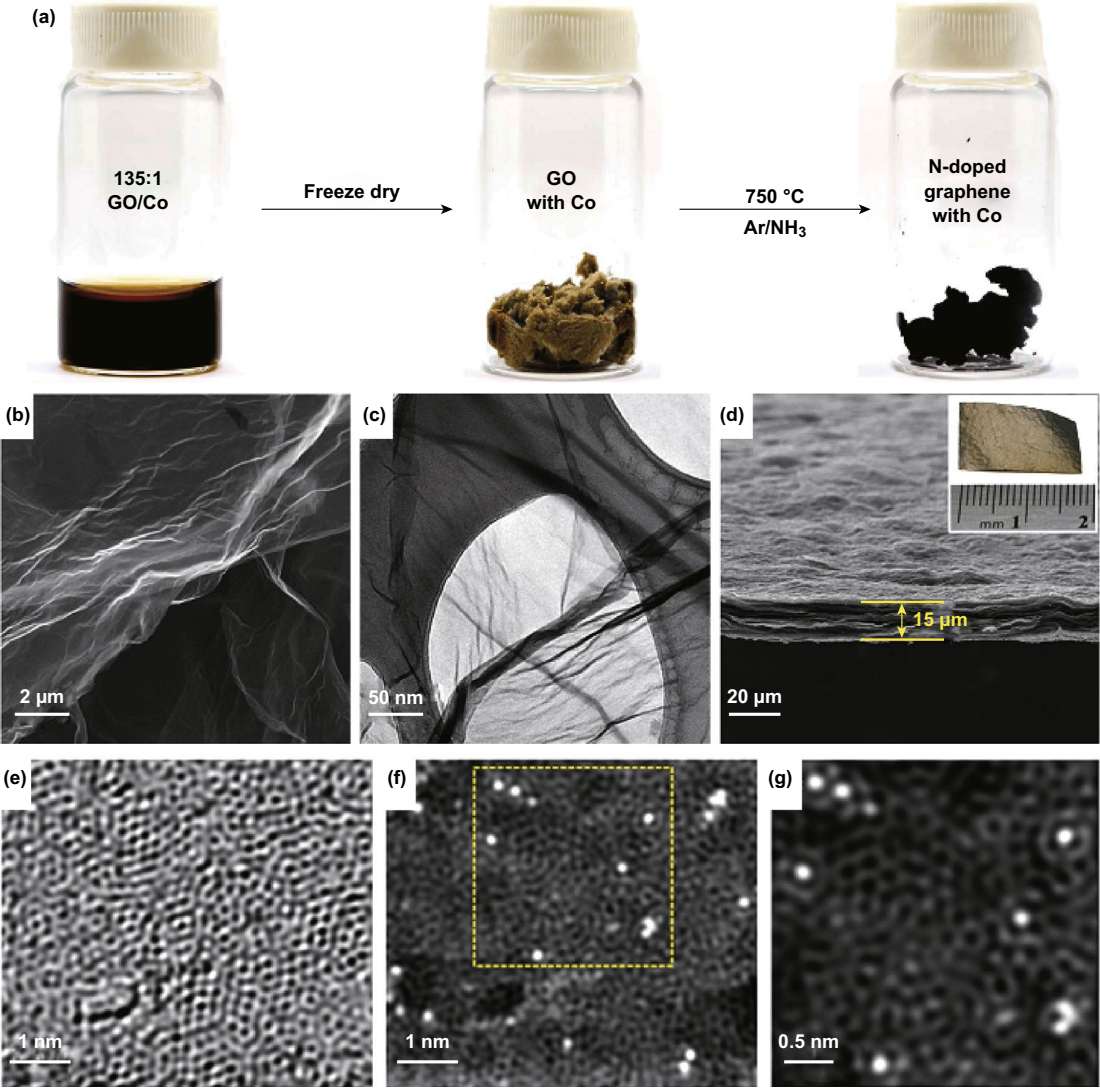
**a** The synthetic procedure of the Co–NG material. **b** SEM, **c** TEM and **d** cross-sectional SEM image of Co–NG nanosheets. **e** Bright-field aberration-corrected STEM image of the Co–NG. **f** HAADF-STEM image of the Co–NG. **g** The enlarged view of the selected area in **f**. Panels are reproduced from Ref. [94] with permission. Copyright 2015 Nature Publishing Group

Figure 8a shows an optical photograph of the preparing process of the nitrogen-doped graphene-supported atomic cobalt (denoted as Co–NG) catalyst. First, the GO and Co salt are dissolved in deionized water. Then the dried sample is obtained by lyophilization. Finally, the Co–NG sample is formed via annealing the dried precursor under the NH_3_ atmosphere. The scanning electron microscopy (SEM) image of Co–NG displays that Co–NG has similar morphological features as graphene (Fig. 8b). Figure 8c further shows Co–NG nanosheets with ripple surface features. In addition, no cobalt nanoparticles can be found on the Co–NG materials. It is worth noting that the Co–NG material can be formed into a paper-like form (Fig. 8d). AC-HAADF-STEM imaging technique was applied to characterization the Co SACs. As shown in Fig. 8e–g, the AC-HAADF-STEM images clearly confirm that Co SACs dispersed on the defects-rich carbon matrix.

### X-Ray Spectroscopy

**Fig. 9:**
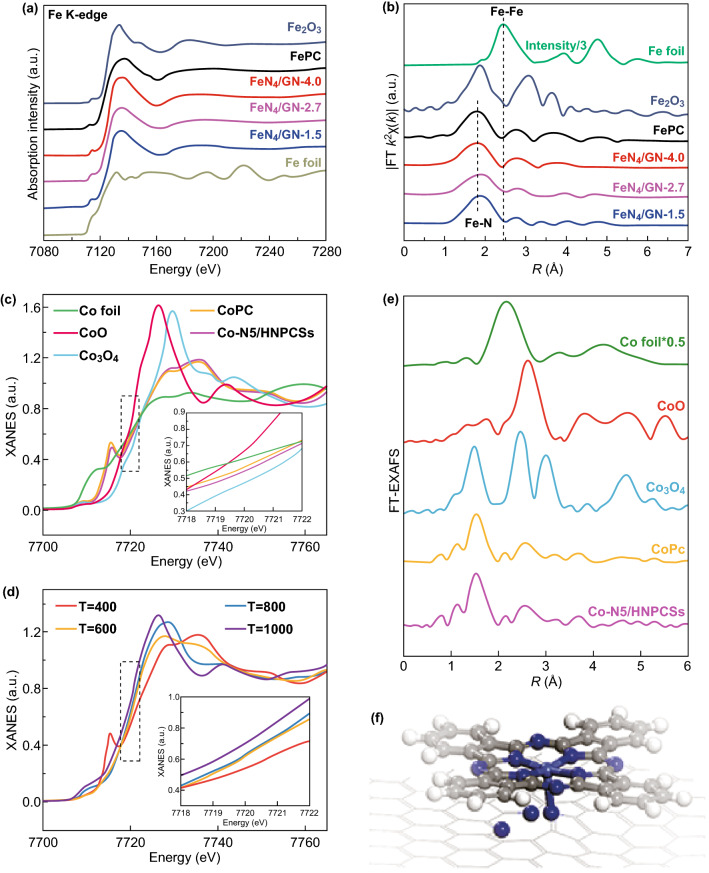
**a** Fe K-edge XANES and **b** FT-EXAFS signals for FeN_4_/GN, FePc, Fe foil, and Fe_2_O_3_. XANES spectra at the Co K-edge of **c** Co_3_O_4_, Co foil, CoO, and Co-N_5_/HNPCSs, **d** Co-N_5_/HNPCSs-T (inset is the magnified image), **e** FT at R space, **f** schematic model, C (gray), N (blue), Co (wathet), and H (white). **a–b** Reproduced from Ref. [78] with permission. Copyright 2017 Wiley-VCH. **c**–**f** Reproduced from Ref. [149] with permission. Copyright 2018 American Chemical Society. (Color figure online)

XANES and EXAFS technologies are widely used for the characterization of the chemical state and the coordination structure of SACs [121]. For example, as presented in Fig. 9a, the Fe K-edge of XANES for FeN_4_/GN samples shows a near-edge structure different from those of Fe_2_O_3_ and Fe foil but similar to that of the iron precursor (FePc), confirming that the valence state of Fe for FeN_4_/GN samples remains the same as that of FePc. The Fourier transform (FT) spectra (Fig. 9b) for FeN_4_/GN samples clearly show that the Fe atoms are atomically dispersed; Fe–Fe bonds are absent [166]. In addition, the atomic Co dopants on the support were also investigated by Pan et al. using XAFS measurements. As shown in Fig. 9c, the Co–K-edge of XANES for Co–N_5_/HNPCSs shows a similar near-edge structure to that of CoPc. Furthermore, the FT k^3^-weighted EXAFS spectra (Fig. 9e) exhibit Co–N bonds with a peak at 1.5 Å, and Co–Co paths at 2.2 Å were not found. When further fitting EXAFS to the quantitative coordination configuration of Co atoms, the Co–N coordination number is five. All of these results demonstrated that the atomic dispersion of Co atoms is formed in Co–N_5_/HNPCSs material. It is worth noting that the Co atomic structure model is illustrated in Fig. 9f (the Co–N coordination number is five) [149].

### Other Complementary Technologies

In addition to AC-HAADF-STEM and XAFS spectroscopy, magic-angle spinning (MAS) NMR and IR are also useful characterization techniques toward SACs. Particularly, solid-state MAS NMR technique is employed to study the anchoring of monoatomic Pt at very low loadings [167]. The coordination-unsaturated penta-coordinated Al^3+^ was confirmed as the anchoring point of Pt SACs on the surface of the γ-Al_2_O_3_ support. Corma et al. [168] also exhibit the presence of Au SACs by the NMR technique. IR spectroscopy can directly detect the interaction between the adsorbed molecule and the surface of the support. The characteristics of the active center can be inferred by appropriate correction by detecting the vibration frequency and intensity of the model. And then the condition of the overall catalyst can be analyzed, which is an important means for characterizing SACs [169, 170].

### DFT Computations

Besides the basic structure characterization, on the one hand, a theoretical study based on DFT has brought unprecedented to predicted catalytic activity through build the structure model, allowing the rational design of materials with tailored performance. For example, by using DFT, Zhou et al. investigated a number of SACs (Ni, Cu, Fe, Co, and Pd) embedded in nitrogen-doped graphenes as both OER and HER catalysts. They concluded that a high-coordinated Co center, e.g., a quadruple-coordinated Co, shows a good OER performance, whereas a low-coordinated Co site, e.g., a triple-coordinated Co, is a good candidate for HER [171]. Likewise, Wang’s group presented a bifunctional single-atom catalyst by DFT simulation. To this end, β_12_-boron monolayer (β_12_-BM)-supported Ni SACs exhibited the best full water splitting performance into the TM_1_/β_12_-BM (TM = Fe, Ti, Co, V, Ni, and Mn) SACs systems [172]. In addition, Ling et al. demonstrated that Mo_1_–N_1_C_2_ possesses ultra-high NRR catalytic activity in a series of SACs of M_1_–N_1_C_2_ (M = Cu, Mo, Pd, and Pt) basis of first-principles computations [120]. On the other hand, understanding the catalytic reaction mechanism is crucial to the rational design of high activity catalysts. Therefore, DFT calculations also have been used to investigate the catalytic reaction mechanism. For example, the hydrogen adsorption free energy (Δ*G*_H*_) is an important descriptor of HER activity. Therefore, the values of Δ*G*_H*_ can be used to compare the HER catalytic activity of different catalysts and generally the closer to zero, the better [147]. Furthermore, researchers can easily understand the local atomic structure and coordination atom information by constructed the DFT model.

## Applications of SACs in Electrochemical HER

**Fig. 10:**
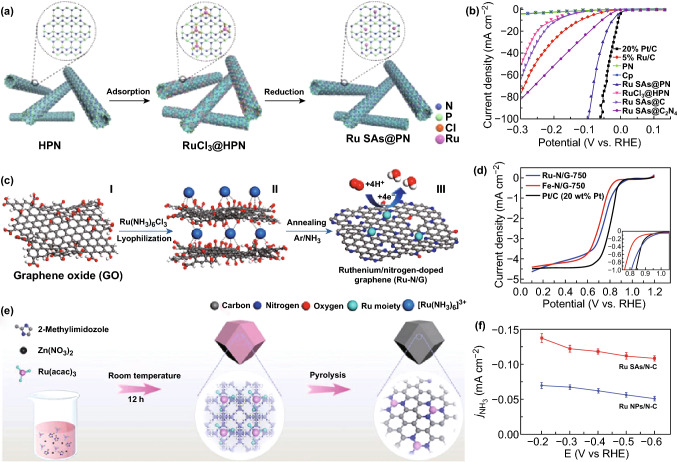
**a** Preparation process of Ru SAs@PN. **b** Polarization curves of the samples. **c** The synthetic process for the Ru–N/G-750. **d** Polarization curves of Ru–N/G-750, Fe–N/G-750 and Pt/C in O_2_-saturated 0.1 M HClO_4_ solutions. **e** Scheme of the synthetic procedure for Ru SAs/N–C. **f** Current densities for NH_3_ production. **a**, **b**, **e**, **f** Reproduced from Refs. [126, 173] with permission. Copyright 2018 Wiley-VCH. **c**, **d** Reproduced from Ref. [106] with permission. Copyright 2017 American Chemical Society

**Fig. 11:**
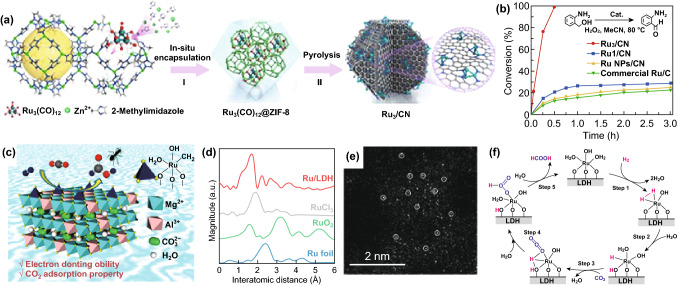
**a** Illustration of the preparation process of Ru_3_/CN. **b** Conversion (%) of 2-aminobenzylalcohol versus time for Ru_3_/CN, Ru_1_/CN, Ru NPs/CN, and commercial Ru/C. **c** Schematic illustration of the hydrogenation. **d** Ru K-edge FT-EXAFS spectra. **e** HAADF-STEM image of Ru/LDH. **f** Possible reaction pathway for CO_2_ hydrogenation to produce formic acid with a Ru/LDH catalyst. Panels are reproduced from Refs. [151, 174] with permission. Copyright 2017 American Chemical Society

As reported, SACs are attractive catalysts that can provide a unique opportunity to tune the catalytic reaction activity and selectivity. For example, Ru SACs have been used as efficient catalysts for HER, ORR, NRR, alcohols oxidation reaction (AOR), and CO_2_ hydrogenation (Figs. 10, 11) [106, 126, 151, 173, 174]. Hydrogen, as a new ideal energy source, is significant to develop fuel cells. Herein, we highlight the recent development of SACs for electrochemical HER applications. HER is a half-reaction that takes place at the cathode during the water splitting. In fact, the HER reaction mechanism is different under acidic and alkaline conditions as represented in Eqs. 1–6:

Acidic media:

1 $$ {\text{Volmer}}\;{\text{reaction:}}\;\;{\text{H}}^{ + } + {\text{e}}^{ - } \to {\text{H}}^{*} $$

2 $$ {\text{Heyrovsky}}\;{\text{reaction:}}\;\;{\text{H}}^{*} + {\text{H}}^{ + } + {\text{e}}^{ - } \to {\text{H}}_{2} $$

3 $$ {\text{Tafel}}\;{\text{reaction:}}\;\;{\text{H}}^{*} + {\text{H}}^{*} \to {\text{H}}_{2} $$

Alkaline media:

4 $$ {\text{Volmer}}\;{\text{reaction:}}\;\;{\text{H}}_{2} {\text{O}} + {\text{e}}^{ - } \to {\text{H}}^{*} + {\text{OH}}^{ - } $$

5 $$ {\text{Heyrovsky}}\;{\text{reaction:}}\;\;{\text{H}}^{*} + {\text{H}}_{2} {\text{O}} + {\text{e}}^{ - } \to {\text{H}}_{2} + {\text{OH}}^{ - } $$

6 $$ {\text{Tafel}}\;{\text{reaction:}}\;\;2{\text{H}}_{2} {\text{O}} + 2{\text{e}}^{ - } \to {\text{H}}_{2} + 2{\text{OH}}^{ - } $$

### Pt Group SACs

**Fig. 12:**
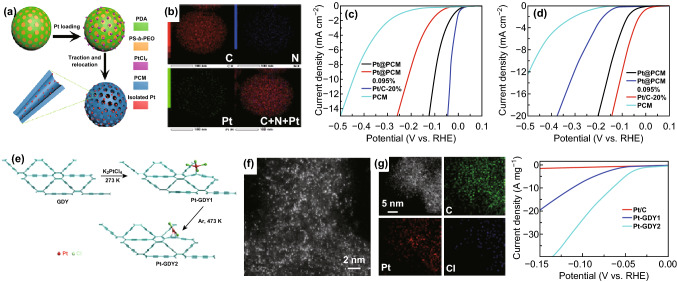
**a** The synthetic process of Pt@PCM. **b** Element mapping images of Pt@PCM. Polarization curves of samples in **c** 0.5 M H_2_SO_4_ and **d** 1.0 M KOH solutions. **e** The formation of Pt-GDY1 and Pt-GDY2. **f** AC-HAADF-STEM images for PtGDY2. **g** Elemental mapping for Pt-GDY2. (h) The polarization curves for Pt-GDY2, Pt-GDY1, and c Pt/C in acidic solution. **a**–**d** Reproduced under the terms of the CC-BY 4.0 license. Ref. [175]. Copyright 2018, American Association for the Advancement of Science. **e**–**h** Reproduced from Ref. [176] with permission. Copyright 2018 Wiley-VCH

Generally, Pt group-based materials are the most active electrocatalysts for HER with large current densities, low overpotential, and good stability. However, they suffer from high cost and scarcity, which limit their extensive application. To solve these problems, many researchers have been carried out to develop low-loading of noble metal HER electrocatalysts. Interestingly, SACs are ideal methods to resolve such problems and are expected to display excellent activity toward HER. For example, Lou’s group reported Pt SACs in a nitrogen-containing porous carbon matrix (Pt@PCM) which can be obtained by a high-temperature atom trapping method (Fig. 12a, b). After HER tests, the Pt@PCM catalyst exhibits 25 times mass activity than that of commercial 20 wt% Pt/C catalyst (Fig. 12c, d). Results of EXAFS investigation and DFT calculations suggested that the active centers are the lattice-confined Pt sites [175]. In addition, Pt SACs dispersed on graphdiyne (GDY) have also been synthesized by the coordination interactions between Pt atoms and alkynyl C atoms in GDY, with the formation of four-coordinated C_2_-Pt-Cl_2_ species (Pt-GDY2). Importantly, Pt-GDY2 shows excellent HER catalytic activity, with an enhanced mass activity in comparison with the 20 wt% commercial Pt/C catalysts (Fig. 12h) [176]. Cheng et al. [52] obtained Pt SACs and clusters supported on the nitrogen-doped graphene nanosheet substrate by the ALD method, which also exhibited high HER activity. Due to the low price of Ru which is 4% cheaper than Pt [106], Ru SACs supported on amorphous phosphorus nitride imide nanotubes (HPN) have also been synthesized by Wu and coworkers. The obtained Ru SACs supported on HPN showed excellent catalytic activity and robust durability in acid solutions toward HER [126].

**Fig. 13:**
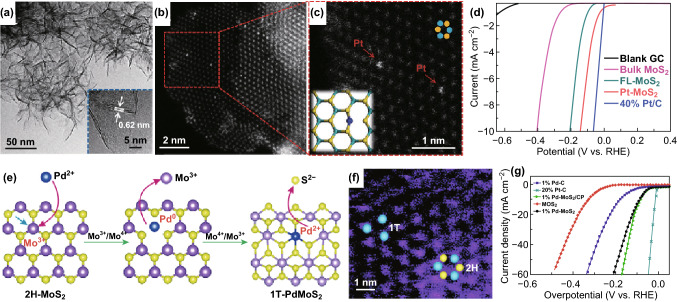
**a** TEM and **b** HAADF-STEM images of Pt–MoS_2_. **c** Magnified domain with red dashed rectangle in **b**. **d** Polarization curves for bulk MoS_2_, FL–MoS_2_, Pt–MoS_2_, and 40% Pt/C. **e** Schematic illustration of the spontaneous MoS_2_/Pd (II) redox reaction. **f** Dark-field STEM image of the 1% Pd–MoS_2_. **g** Polarization curves of 1% Pd–MoS_2_/CP, 1% Pd–MoS_2_, pure MoS_2_, 1% Pd–C, and 20% Pt/C. **a**–**d** Reproduced from Ref. [92] with permission. Copyright 2015 Royal Society of Chemistry. **e**–**g** Reproduced from Ref. [177] with permission. Copyright 2018 Nature Publishing Group

Traditionally, transition metal phosphides (TMPs) and chalcogenides (TMCs) have been widely studied as HER electrocatalyst due to their excellent activity. Therefore, SACs supported on TMPs and TMCs are expected to show outstanding HER performance. Indeed, Luo’s group fabricated Pt SACs on CoP nanotube arrays (Pt SACs/CoP) through the electrodeposition method. The Pt SACs/CoP (Pt loading: 1.76 wt%) shows better HER activity than that of commercial 20 wt% Pt/C [53]. Deng et al. also prepared Pt SACs (Fig. 13a–c) uniformly dispersed on MoS_2_ (Pt–MoS_2_), showing significantly boosted HER activity compared with original MoS_2_ (Fig. 13d) [92]. In addition, Xing and coworkers fabricated different loading of single Pd atoms doped MoS_2_ (Fig. 13e, f). The 1% Pd–MoS_2_ shows the best HER catalytic activity with a low overpotential of 89 mV at a current density of 10 mA cm^−2^ (Fig. 13g) [177]. Guan et al. [178] designed and prepared special ganoderma-like MoS_2_/NiS_2_ heterostructures with dispersed Pt atoms which showed impressive HER performance. Despite such progress on the Pt group SACs, their large-scale commercialization is still hindered by their scarcity and high cost. Thus, it is worthwhile to develop non-noble metal-based SACs due to their relatively abundant and cheaper resource.

### Fe-, Co-, Ni-Based SACs

**Fig. 14:**
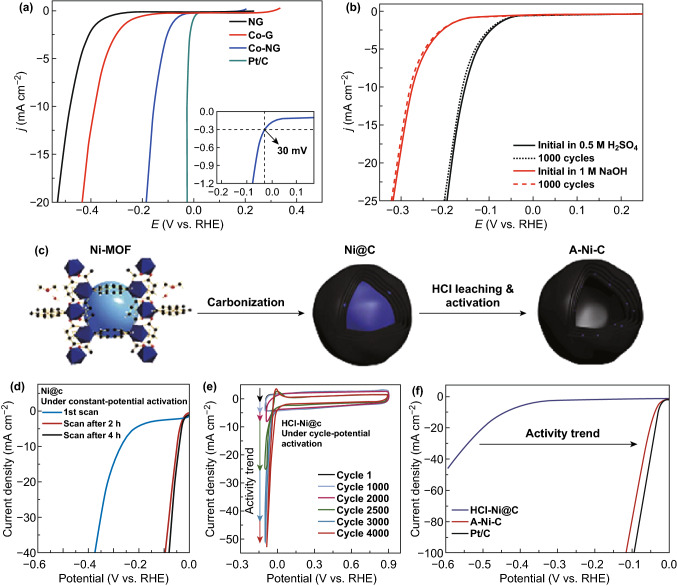
**a** The polarization curves of Co–G, NG, Co–NG and commercial Pt/C in acidic solutions. **b** Polarization curves for the Co–NG before and after 1000 CV cycles in acidic and alkaline solutions, respectively. **c** The preparation and activation process of the Ni-C materials. **d** Polarization curves before and after activation at a constant potential. **e** CVs of HCl-Ni@C. **f** Polarization curves for A–Ni–C, HCl-Ni@C, and Pt/C catalysts. Panels are reproduced from Refs. [94, 179] with permission. Copyright 2015 and 2016 Nature Publishing Group

**Fig. 15:**
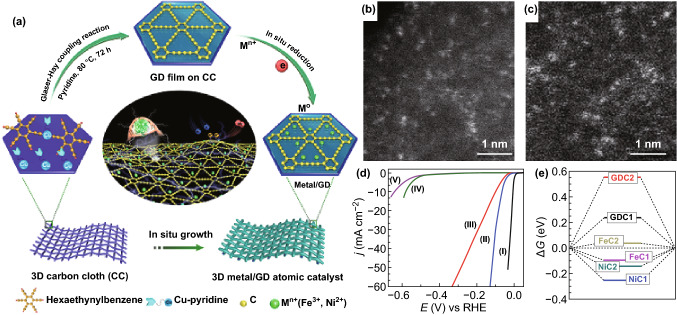
**a** Schematic illustration for the synthesis of Fe/GD and Ni/GD. AC-HAADF-STEM images of **b** Ni/GD and **c** Fe/GD. **d** Polarization curves of CC, Ni/GD, GDF, Fe/GD, and Pt/C. **e** The chemisorption energy of *H* for HER performance related to the free energy profile (Δ*G*). Panels are reproduced from Ref. [155] with permission. Copyright 2018 Nature Publishing Group

Tour’s group reported a low-cost, simple, and scalable method to preparing Co SACs by simply heat-treating cobalt salts and graphene oxide under NH_3_ atmosphere for the first time. The Co–NG catalysts exhibit excellent HER activity under both acidic and alkaline conditions (Fig. 14a, b). They also suggested that the catalytically active centers originate from Co metal centers coordinated to the N atoms [95]. In addition, Fan et al. [179] reported that a Ni–C-based material can be activated to obtain the Ni SACs on graphitic carbon after 4000 cyclic voltammograms cycles, consequently displaying high catalytic activity and durability for HER (Fig. 14c–f). Similarly, Chen’s group developed Ni SACs embedded in nanoporous graphene which also exhibited superior catalytic activities and stability in water splitting reactions under acidic conditions [180]. Fe SACs have also been widely reported by different researchers, because element Fe is one of the most abundant and cheapest transition metals [150, 155, 166, 181]. For example, recently, Xue et al. reported a facile and precise anchoring of the Fe SACs on graphdiyne (Fe/GD) by electrodeposition method using Fe^3+^ and graphdiyne as a precursor (Fig. 15a). Fe/GD exhibits high HER activity with only 66 mV overpotential at 10 mA cm^−2^ (Fig. 15d). Additionally, the atomic-level identification of the active structure during the alkaline HER has been reported recently. They demonstrated that the formation of a high-valence HO–Co_1_–N_2_ moiety by the binding between isolated Co_1_–N_4_ sites with electrolyte hydroxide further unravels the preferred water adsorption reaction intermediate H_2_O–(HO–Co_1_–N_2_). This result is critical to industrial water–alkali electrolyzers, which remains elusive and is a field of intense research [182].

### Other Transition Metal-Based SACs

**Fig. 16:**
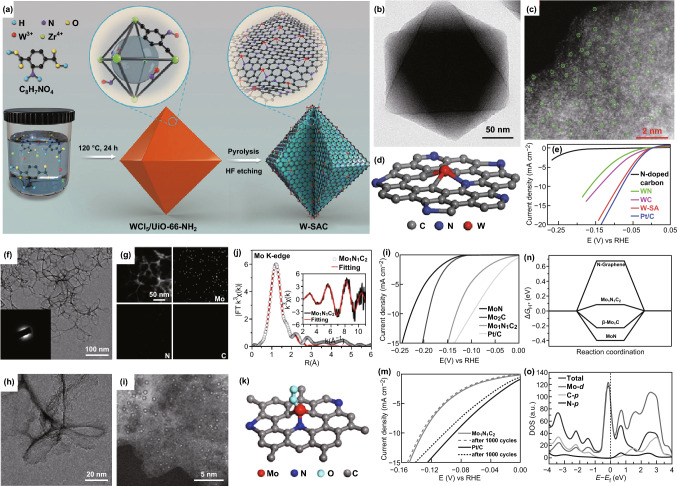
**a** The formation process of W SACs. **b** TEM and **c** AC-HAADF-STEM images of the W SACs. **d** Atomic structure model of the W SACs. **e** Polarization curves for the W SACs. **f** TEM image of the Mo_1_N_1_C_2_, the insert is SAED pattern. **g** EDS maps for Mo_1_N_1_C_2_ material. **h** HRTEM and **i** AC-HAADF-STEM images of the Mo_1_N_1_C_2_. **j** Mo K-edge FT-EXAFS fitting curves of the Mo_1_N_1_C_2_. **k** Atomic structure model of the Mo_1_N_1_C_2_. **l** Polarization curves for the Mo_1_N_1_C_2_, MoN, Mo_2_C, and 20% Pt/C. **m** Polarization curves for the Mo_1_N_1_C_2_ before and after 1000 CV cycles. **n** Gibbs free energy for H* adsorption for Mo_2_C, Mo_1_N_1_C_2_, and MoN. **o** The calculated DOS of the Mo_1_N_1_C_2_. Panels are reproduced from Refs. [148, 153] with permission. Copyright 2018 and 2017 Wiley-VCH

Traditionally, Mo- and W-based materials are also excellent catalysts for HER. Therefore, it is interesting to investigate the HER activity of Mo and W SACs. Indeed, Wang and coworkers demonstrated that W SACs with high HER performance can be obtained by the MOF-derived method (Fig. 16a, e). HAADF-STEM and XAFS spectroscopy analyses show the formation of W SACs anchored with three C atoms and one N atom (W_1_N_1_C_3_) (Fig. 16b–d). DFT calculations further demonstrated that the unique structure of the W_1_N_1_C_3_ moiety plays a critical role in improving HER activity [148]. Additionally, Mo SACs were obtained with the help of chitosan by the same group. Similarly, the structure of Mo SACs was probed by XAFS spectroscopy and AC-HAADF-STEM, suggesting the formation of Mo SACs anchored with two carbon atoms and one nitrogen atom (Mo_1_N_1_C_2_) (Fig. 16f–k). The Mo_1_N_1_C_2_ material displays outstanding HER performance compared with bulk MoN and Mo_2_C and better durability than 20 wt% commercial Pt/C (Fig. 16l, m). DFT calculations further reveal that Mo_1_N_1_C_2_ is more beneficial for HER electrocatalysis than MoN and Mo_2_C (Fig. 16n, o) [154]. Recently, Du’s group demonstrated that V SACs supported on graphene is also a highly active HER electrocatalyst through DFT calculations [183]. It was suggested that V SACs embedded in the graphene can substantially tune the free energy of hydrogen adsorption (Δ*G*_H*_) to a more optimal value (Δ*G*_H*_ = − 0.01 eV), which is even better than the Pt material.

It is worth noting that within the last few years there has been a fast growth in the study fields of SACs. All kinds of noble and non-noble SACs have been fabricated and used as a high activity HER electrocatalysts. Table 2 lists a detailed comparison of their electrochemical HER performances.

**Table 2:** Summary of the HER activity of SACs in acid/neutral/alkaline solutions

Catalysts	Electrolytes	*η*@*j* (mV@mA^−2^)	Tafel slope (mV dec^−1^)	References
Pt–MoS_2_	0.1 M H_2_SO_4_	~ 150@10	96	[92]
ALD50Pt/NGNs	0.5 M H_2_SO_4_	50@16	29	[52]
400-SWMT/Pt	0.5 M H_2_SO_4_	27@10	38	[93]
Pt-GDY2	0.5 M H_2_SO_4_	~ 50@30	38	[176]
PtSA-NT-NF	1.0 M PBS	24@10	30	[53]
Pt SAs/DG	0.5 M H_2_SO_4_	23@10	25	[160]
Mo_2_TiC_2_T_*x*_-Pt_SA_	0.5 M H_2_SO_4_	30@10	30	[161]
Pt@PCM	0.5 M H_2_SO_4_	105@10	65.3	[175]
Pt@PCM	1.0 M KOH	139@10	73.6	[175]
Pt_1_–MoO_3**−***x*_	0.5 M H_2_SO_4_	23.3@10	28.8	[185]
Pt SASs/AG	0.5 M H_2_SO_4_	12@10	29.33	[186]
SANi-PtNWs	1.0 M KOH	70@10	60.3	[187]
Pt1/NMC	0.5 M H_2_SO_4_	55@100	26	[188]
Pt/np-Co_0.85_Se	1.0 M PBS	55@10	35	[189]
Pd–MoS_2_	0.5 M H_2_SO_4_	78@10	62	[177]
Pd/Cu–Pt NRs	0.5 M H_2_SO_4_	22.8@10	25	[190]
Ru SAs@PN	0.5 M H_2_SO_4_	24@10	38	[126]
Ru@Co SAs/N–C	1.0 M KOH	7@10	30	[191]
Ru–MoS_2_/CC	1.0 M KOH	41@10	114	[192]
Pt–Ru dimer	0.5 M H_2_SO_4_	~ 20@10	28.9	[193]
Fe/GD	0.5 M H_2_SO_4_	66@10	37.8	[155]
Ni/GD	0.5 M H_2_SO_4_	88@10	45.8	[155]
A–Ni–C	0.5 M H_2_SO_4_	34@10	41	[179]
Ni-doped graphene	0.5 M H_2_SO_4_	180@10	45	[180]
A-Ni@DG	0.5 M H_2_SO_4_	70@10	31	[184]
SANi-I	1.0 M KOH	60@100	34.6	[194]
Co–NG	0.5 M H_2_SO_4_	147@10	82	[94]
Co_1_/PCN	1.0 M KOH	138@10	52	[182]
Co SAs/PTF-600	0.5 M H_2_SO_4_	94@10	50	[195]
Mo_1_N_1_C_2_	0.5 M H_2_SO_4_	154@10	86	[153]
W_1_N_1_C_3_	0.5 M H_2_SO_4_	105@10	58	[148]

## Summary and Outlook

Single-atom catalysts (SACs), with maximum atom-utilization efficiency, exhibit many advantages particularly for HER application with high activity and stability. In recent years, intensive researches have been carried out in this field. In this review, all the important SACs synthetic strategies reported so far, including the wet-chemistry method, atomic layer deposition (ALD), metal–organic framework (MOF)-derived method, electrodeposition, high-temperature atom trapping from bulk particles, and vacancies/defects immobilized strategy, have been included and discussed in detail. In addition, to reveal the structures and compositions of SACs, various advanced characterization techniques, such as aberration-corrected high-angle annular dark-field scanning transmission electron microscopy (AC-corrected HAADF-STEM), X-ray absorption near-edge structure (XANES), and extended X-ray absorption fine structure (EXAFS) techniques as well as density functional theory (DFT) simulation have also been summarized and discussed. Finally, various metal-based (especially Pt, Pd, Ru, Fe, Co, Ni, Mo, W) SACs in electrocatalytic HER have been systematically reviewed. Despite these significant achievements in the past few years in SACs for electrochemical HER field, there are still challenges in this fascinating field that remain to be resolved:

1. As it is known, SACs need to be dispersed on support to avoid prevent them from aggregation and at the same time to increase their utilization efficiency during the catalytic reactions. Therefore, supports play an important role in SACs synthesis and their catalytic processes. To this end, more efforts should be devoted to finding new, more conductive and robust support materials.
2. At present, only Pt, Pd, Ru, Fe, Co, Ni, Mo, W SACs have been investigated in the HER catalytic field. Based on experimental evidence, V, Nb, Ta, Mn, Rh, Ir, Ag, Au, and Cu metal compounds have also been reported to be potential electrocatalysts for HER. Therefore, research efforts should be extended to more metal SACs for HER.
3. To investigate the active centers and understand the catalytic reaction mechanism, advanced in situ and ex situ characterization techniques are highly desirable and beneficial for the rational design and observation of high-performance SACs.
4. DFT simulation is a useful tool in disclosing the catalytic reaction mechanism and predicting the catalytically active species. However, accurate assessment of the catalytic centers resulting from SACs electrocatalysts still needs further in-depth study.
5. Until now, the electrocatalytic behavior of SACs is still ambiguous. Consequently, DFT simulations in combination with XAFS fitting and the experimental data have brought unprecedented insight into the real mechanism.
6. Although most of SACs exhibit Pt-like or even better than Pt activity toward HER, it should be pointed out that the durability problem is another huge challenge for SACs toward practical application; particularly, in industrial polymer electrolyte membrane (PEM) electrolyzers always need catalysts to maintain high activity and stability over 50,000 h.

Overall, the future directions of SACs development for HER can be focused on developing new and more efficient SACs and their supporting materials, discovering the HER catalytic reaction mechanisms at the molecular scale by a combination of experimental result and DFT calculations, and exploring new SACs for HER and their structural analysis.
